# Long‐term stability of a 50‐kV X‐ray unit for stereotactic irradiation

**DOI:** 10.1120/jacmp.v7i3.2238

**Published:** 2006-08-24

**Authors:** Peter J. Biggs

**Affiliations:** ^1^ Radiation Oncology Department Massachusetts General Hospital Fruit Street Boston Massachusetts 02114 U.S.A.

**Keywords:** low‐energy X‐rays, scintillation counters, stereotactic irradiation

## Abstract

A low‐energy (50 kV) X‐ray tube used for the stereotactic irradiation of intracranial lesions has been in use since 1999. The unit is calibrated prior to every procedure and during periodic quality assurance (QA) tests. The unit uses an internal and an external scintillation detector to monitor dose as well as a conventional timer. The records of these calibrations were reviewed to see whether a change in the output had occurred over that period. Using time as the reference, it was found that both the internal radiation monitor (IRM) and the beam output, determined with a parallel plate ionization chamber, dropped by variable amounts over the given period. The beam output dropped significantly more than the IRM, while the external radiation monitor (ERM) showed no significant deviation from its initial value. The beam output dropped to about 90% of its initial value after about 200 days but remained relatively constant thereafter. The IRM dropped steadily to about 96% to 97% of its initial value at 1000 days, but recovered to about 98% after that. Calibration prior to each procedure is strongly recommended.

PACS numbers: 87.53.Dq; 87.56.By

## I. INTRODUCTION

An interstitial radiosurgery source (Carl Zeiss Surgical, GmbH, Germany (formerly Photoelectron Corporation)) has been in clinical use at the Massachusetts General Hospital (MGH) since 1992. The prototype and earlier version (Mark III) both used a tube that suffered some outgassing due to the method of combining the electron gun with the tube, as a result of which, the output declined not only with time, but also during the course of a treatment. This necessitated making corrections to the treatment time to account for this decay.

The Intrabeam (formerly known as the PRS400) was a much improved, commercial version that eliminated this problem, and a unit has been in use at MGH since June 1999. This device has been used predominantly for the treatment of intracranial lesions (brain metastases, primary glioma, and meningioma following surgical resection), but there is significant interest in its use for the treatment of breast cancer (Vaidya et al.^(1)^), both in the United States and Europe where clinical trials are underway. The original X‐ray tube in this unit was replaced in August 2000, and this replacement tube has been in operation ever since (August 2005). The Intrabeam is equipped with an internal radiation monitor (IRM), which is an onboard monitor used to set the length of treatment (much like the ion chambers on a LINAC), and an external radiation monitor (ERM), which is an independent device placed near the source. Both the ERM and the IRM are scintillation detectors, and the readings are recorded in counts. The IRM is used to determine the length of treatment, not the timer, and the ERM is used as a backup. Although the ERM has no absolute calibration since its location relative to the treatment site and intervening tissue is variable, the control system samples the count rate and uses that as a basis for extrapolating the effective termination count.

Energies of 30 kV, 40 kV, and 50 kV and beam currents of 5 μA, 10 μA, 20 μA, and 40 μA can be selected. Absolute calibration of the device is carried out in water. However, since water calibration cannot be performed in the OR (the equipment has to remain sterile for the treatment), calibration is also carried out in air, since this procedure can be performed in the OR under sterile conditions. Data from these calibrations have been accumulated since August 2000, and the purpose of this paper is to examine the long‐term effects of use on the output and the IRM and ERM monitors. Over the period in question, a patient has been treated about once a month, on average. Details of the commissioning of the original device have been published elsewhere by Beatty et al.^(2)^

## II. METHODS AND MATERIALS

### A. Equipment

**Figure 1:**
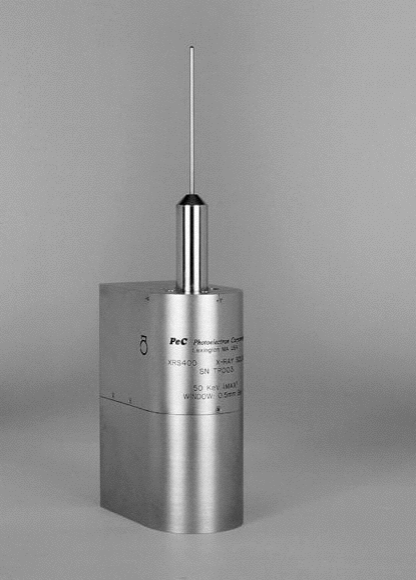
The Intrabeam XRS unit. The beam is produced at the tip of the 3.5‐mm diameter tube and is approximately isotropic.

**Figure 2:**
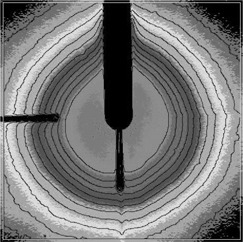
Isodose curves for the XRS unit. Centering of the isodoses around the tip of the probe is clear. Note that the isodose curves are pulled out just around the tube. These curves were taken with radiochromic film with the axis of the tube in the plane of the film.

Figure 1 shows the X‐ray unit. The X‐rays are produced at the tip of a 10‐cm long, 3.5‐mm diameter tube by electrons striking a thin, gold target. The thick cylinder at the base of the tube contains steering coils to center the beam on the target. The base houses the gun, high‐voltage electronics, and the IRM. Its overall weight is about 2 kg. The X‐rays are produced almost isotropically except in the backward direction, where the isodose curves are stretched toward the source, probably due to backward scattered photons traveling in the vacuum in the tube, unattenuated by the water phantom. Figure 2 shows a series of isodose curves obtained from exposing radiochromic film to 40‐kV X‐rays. The contour values were arbitrarily chosen to illustrate the shape of the dose distribution.

**Figure 3:**
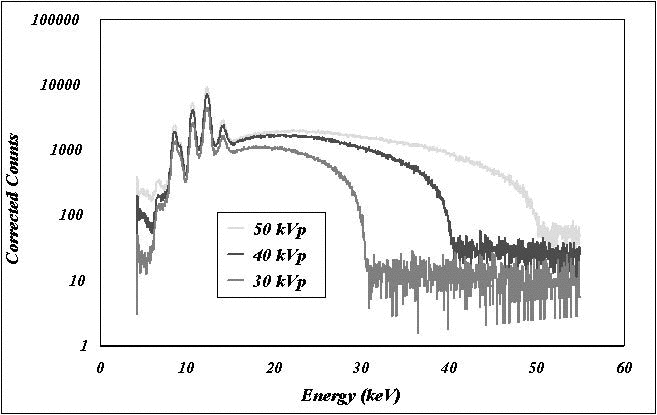
Spectra for the Intrabeam XRS unit for 30 kV, 40 kV, and 50 kV in air. Note the sharp characteristic peaks at low energies (see text for details). The broad hump is due to *bremsstrahlung* X‐rays.

The X‐ray unit was originally devised to fit into a stereotactic head frame to center the tip of the device at the same point where the biopsy is taken (center of the tumor). The ERM is inserted in one of the stanchions on either side of the head frame, depending on which side of the head the lesion is located. Figure 3 shows the energy spectrum for the three energies measured with a Si(Li) detector (Model 3000, KeVex, Burlingame, CA). The highest three peaks at low energy correspond to the characteristic L lines of the gold target. The lowest peak derives from the K line of nickel in the biocompatible coating. The broad hump that stretches to the peak energies corresponds to *bremsstrahlung* radiation.

Since the unit is calibrated prior to every procedure and on other occasions for quality assurance (QA) purposes, there is a documented record of the output of the device, both through the IRM and the X‐ray output, as measured with an ionization chamber. Typically, the pretreatment calibration is carried out only at 50 kVp for one or two currents (40 μA and 20 μA), but during QA work, all energies (50 kV, 40 kV, and 30 kV) and currents (5 μA, 10 μA, 20 μA, and 40 μA) are tested. Note, however, that every time the device is calibrated, both the IRM and the ERM are measured at all beam energies and beam currents.

The ionization chamber used was a thin‐window, parallel plate chamber, with a 0.03‐mm thick polyethylene window and a volume of 0.02 cm^3^ (PTW, model 30‐334, Freiburg, Germany). The calibration of this chamber is checked annually against a secondary standard (Accredited Dosimetry Calibration Laboratory). In the in‐air output calibrations, the chamber is in a fixed geometry with respect to the source. A shielded housing slides snugly over the base of the tube. The ionization chamber is inserted into a slot in this housing with its surface orthogonal to the axis of the tube (end on); when in position, a steel flap covers the chamber to prevent leakage radiation. The in‐water measurements were made in a dedicated water tank. Measurements were made of the distance–radius dose from 3 mm to 20 mm. The output of the device is defined as the dose in water at 10 mm from the target. This distance is accurate to about 0.1 mm to 0.2 mm. Details of the distance calibration are given in Ref. 2. For the results reported here, however, only the in‐air measurements were used. Note, also, that the ERM readings reported here are also taken under fixed geometry conditions (equipment not shown). For the purpose of this paper, X‐ray output is synonymous with ionization chamber beam current and is referred to as “beam output.”

### B. Analysis

For each energy and beam current, each data point was normalized to the date when the present device was first used. Plots were made of the beam, IRM and ERM outputs for each beam energy, and output current. These plots were examined to see if there were any significant differences in the outputs as a function of beam current. These outputs were then averaged over all four beam currents and the plots at the three energies compared. Finally, the average of all the readings (beam current, beam energy) was taken for these output parameters. In some cases, this average included all beam currents and energies, but in many cases it was averaged at most over one beam energy with between 2 and 4 beam currents. About 25% of the calibrations included all points, and approximately 70% included four or more points. Plots of change with time were made for output (beam current), IRM, ERM, and output (beam current)/IRM. The variation in the IRM, ERM, and ion chamber current readings was examined over a period of approximately 5 years (1800 days).

## III. RESULTS AND DISCUSSION

**Figure 4:**
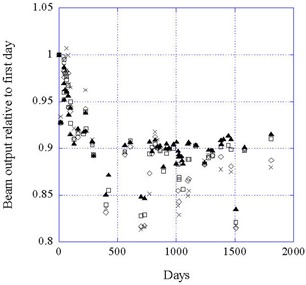
Data for the beam output at 50 kV for four beam currents as a function of time. (▲) 40 μA; (□) 20 μA; (◊) 10 μA; (X) 5 μA.

**Figure 5:**
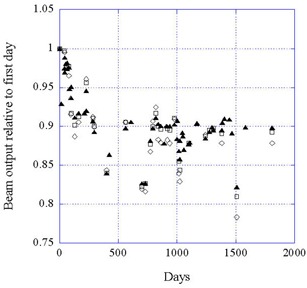
Data for the beam output at 50 kV, 40 kV, and 30 kV, each averaged over the four beam currents, as a function of time. (▲) 50 kV; (□) 40 kV; (▪) 30 kV.

Figure 4 shows the beam output for all four beam currents at 50 kV. It can be seen that the data are consistent for all currents, as would be expected since the output is linear with current. When averaged over all currents, the data for the three energies show no significant differences, as shown in Fig. 5.

**Figure 6:**
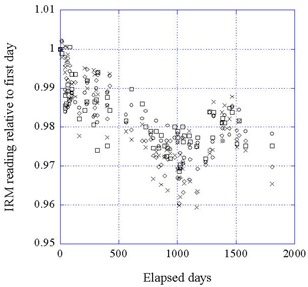
Data for the IRM at 50 kV and 40 kV for 40 mA and 10 mA, as a function of time. (○) 50/40; (□) 50/10; (◊) 40/40; (x) 40/10. (Note: The notation X/Y here means X RS voltage (kV)/beam current (μA).)

Figure 6 shows the data for the IRM for two beam currents at 50 kV and 30 kV. The 40‐kV data and the data for the other currents have been omitted for clarity. Inspection of the full dataset shows that the data are consistent for all beam energies and currents except for the lowest energy (30 kV) at the two lowest currents (5 μA and 10 μA), where the spread is much greater. The reason for this is probably that the number of counts for these two settings is low, the averages being 272 and 544, respectively.

**Figure 7:**
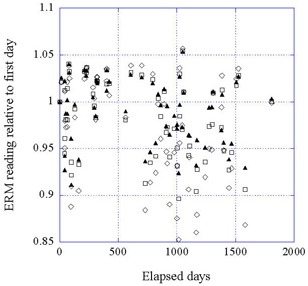
Data for the ERM at 50 kV, 40 kV, and 30 kV, each averaged over the four beam currents, as a function of time. (▲) 50 kV; (□) 40 kV; (◊) 30 kV.

Figure 7 shows the data for the ERM at 50 kV, 40 kV, and 30 kV, averaged over all beam currents. There is also consistency between the datasets, primarily due to the fact that there is no significant trend in the relative value over the whole time period, although the latitude is wide, from +5% to −10%. The data at 50 kV may have a slightly smaller spread compared to the other energies

**Figure 8:**
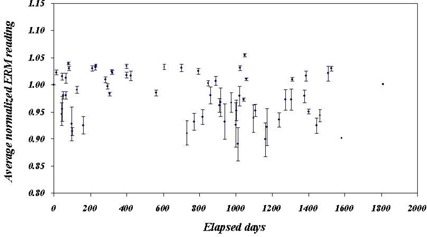
Average normalized ERM reading as a function of time. The error bars indicate the variation in the readings for each calibration.

**Figure 9:**
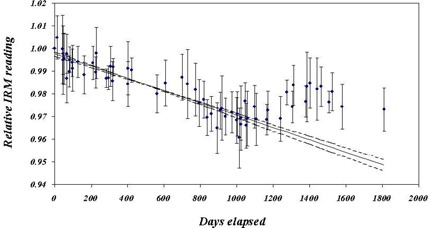
Average normalized IRM as a function of time. The error bars indicate the variation in the readings for each calibration. The lines indicate fits to the data for the first 100 days. The solid line is the best fit, and the dashed lines indicate the upper and lower limits, as indicated by the error on the fit.

**Figure 10:**
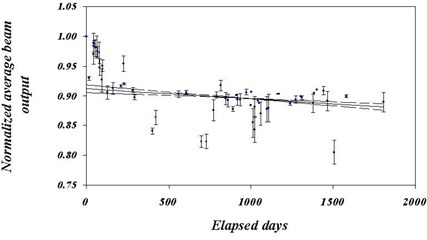
Average normalized beam output as a function of time. The error bars indicate the variation in the readings for each calibration. The lines indicate fits to the data after day 128 (after the initial drop), but excluding the outliers at about days 450, 750, and 1500. Since this fit is merely to guide the eye, there is no loss in dropping these points. The solid line is the best fit, and the dashed lines indicate the upper and lower limits, as indicated by the error on the fit.

Figures 8, 9, and 10 show the normalized outputs, averaged over all energy and beam current readings, for the ERM, IRM, and beam output, respectively. Plots include error bars indicating the variation between the points. Whereas the ERM output was clearly constant over this period, both the IRM and X‐ray output declined with time, the X‐ray output considerably more than the IRM. The constancy of the ERM output is to be expected since the ERM is used less frequently and is much farther from the source than the IRM. The drop in IRM output is characterized by a constant decline (3% over 1000 days), after which it is statistically flat. The lines correspond to regression fits to the data over the range 0 to 1000 days. The solid line is the best fit, and the dashed lines correspond to the uncertainty in the slope. The drop in the X‐ray output is more precipitous and uneven, dropping about 10% over the first 200 days. After that, the X‐ray output is relatively constant to 1800 days at ~90% of initial output. Here, also, the lines are regression fits to the data, with the solid and dashed lines having the same meaning. In this case, the fit is taken approximately between day 250 and day 1800, with the exception of the low‐lying outliers. This exclusion is acceptable, since the purpose of the fit is only to obtain a sense of the general trend of the data. The data would seem to indicate that the X‐ray tube started with a period of adjustment, a “wearing‐in” process, after which the output is relatively stable.

**Figure 11:**
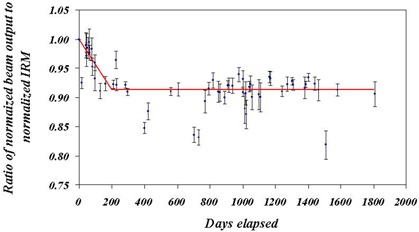
Ratio of average beam output to IRM as a function of time. The error bars indicate the variation in the readings for each calibration. This plot shows that the beam output as measured by the ionization chamber and the IRM do not track each other. The plot closely resembles the beam output data in Fig. 9.

Figure 11 shows the ratio of the X‐ray output to the IRM counts as a function of time. The lines are not fits but are meant to guide the eye to emphasize the drop in the ratio over the first 200 days, followed by a flat response, again with the exception of the outliers. Since the curve drops below unity, it can be assumed that the drop in X‐ray output exceeds the drop in response of the IRM. However, the curve is statistically quite flat beyond 200 days. It also means that the IRM is changing independent of the X‐ray output. One might suggest that this drop in the ratio of beam output to the IRM reading implies that the dose calibration has changed by ~10% over the time period. This is definitely not the case, however, since prior to every procedure, the IRM and ERM readings are measured along with the X‐ray output in water and in air. Even for a 9% change in the beam output/IRM ratio over 200 days, the change over 1 day, the maximum delay between calibration and treatment, is <0.05%.

## IV. CONCLUSIONS

The change in output and calibration of an Intrabeam X‐ray device has been monitored over a 5‐year period. Three independent systems are used to monitor the X‐ray output: an internal radiation monitor (IRM), an external radiation monitor (ERM), and an ion chamber to measure the X‐ray output. As expected, the ERM did not change with time, but both the IRM and the X‐ray output dropped, the IRM by 3% and the X‐ray output by about 10% over the period of observation. Constant calibration of the device ensures that accurate dose delivery is achieved. These data apply to the tube investigated. Although one would expect other tubes to show a decline in output and IRM efficiency, their declines would not necessarily mimic the data presented here. That would depend on factors such as frequency of use and environmental conditions. For example, the manufacturer cautions against immersing the bare probe in water since it will damage the surface, which, in turn, will affect the X‐ray output.
